# 5D solid-state NMR spectroscopy for facilitated resonance assignment

**DOI:** 10.1007/s10858-023-00424-5

**Published:** 2023-11-09

**Authors:** Alexander Klein, Suresh K. Vasa, Rasmus Linser

**Affiliations:** Department of Chemistry and Chemical Biology, TU Dortmund University, Otto-Hahn-Str. 4a, 44227 Dortmund, Germany

**Keywords:** Solid-state NMR, Proton detection, Fast magic-angle spinning, Higher dimensionality, 5D, Resonance assignment, Minimal set of experiments, Non-uniform sampling

## Abstract

^1^H-detected solid-state NMR spectroscopy has been becoming increasingly popular for the characterization of protein structure, dynamics, and function. Recently, we showed that higher-dimensionality solid-state NMR spectroscopy can aid resonance assignments in large micro-crystalline protein targets to combat ambiguity (Klein et al., Proc. Natl. Acad. Sci. U.S.A. 2022). However, assignments represent both, a time-limiting factor and one of the major practical disadvantages within solid-state NMR studies compared to other structural-biology techniques from a very general perspective. Here, we show that 5D solid-state NMR spectroscopy is not only justified for high-molecular-weight targets but will also be a realistic and practicable method to streamline resonance assignment in small to medium-sized protein targets, which such methodology might not have been expected to be of advantage for. Using a combination of non-uniform sampling and the signal separating algorithm for spectral reconstruction on a deuterated and proton back-exchanged micro-crystalline protein at fast magic-angle spinning, direct amide-to-amide correlations in five dimensions are obtained with competitive sensitivity compatible with common hardware and measurement time commitments. The self-sufficient backbone walks enable efficient assignment with very high confidence and can be combined with higher-dimensionality sidechain-to-backbone correlations from protonated preparations into minimal sets of experiments to be acquired for simultaneous backbone and sidechain assignment. The strategies present themselves as potent alternatives for efficient assignment compared to the traditional assignment approaches in 3D, avoiding user misassignments derived from ambiguity or loss of overview and facilitating automation. This will ease future access to NMR-based characterization for the typical solid-state NMR targets at fast MAS.

**Supplementary Information:**

The online version contains supplementary material available at 10.1007/s10858-023-00424-5.

## Introduction

Owing to deuteration strategies (Akbey et al. 2009; Chevelkov et al. 2006; Linser et al. 2011b; Reif 2021; Vasa et al. 2018a) and facilitated by increasing Magic-Angle-Spinning (MAS) frequencies (Cala-De Paepe et al. 2017; Lewandowski et al. 2011; Schledorn et al. 2020), effectively reducing the influence of coherent contributions from proton-proton dipolar couplings (Böckmann et al. 2015; Malär et al. 2019; Penzel et al. 2019), proton detection has been facilitating effective resonance assignment (Andreas et al. 2015; Barbet-Massin et al. 2013; Klein et al. 2022a, b; Knight et al. 2011, 2012; Linser et al. 2010a, b; Orton et al. 2020; Schubeis et al. 2021; Stanek et al. 2016, 2020; Vasa et al. 2019; Xiang et al. 2016; Zhou et al. 2007c), assessment of protein dynamics with timescales overarching many orders of magnitudes (Chevelkov et al. 2007, 2009; Gauto et al. 2017; Grohe et al. 2020; Kurauskas et al. 2017; Ma et al. 2014; Rovó 2020; Rovó and Linser 2018; Rovó et al. 2019; Schanda and Ernst 2016; Singh et al. 2019; Vasa et al. 2018a), as well as protein structure elucidation (Andreas et al. 2016; Bertarello et al. 2020; Grohe et al. 2019; Huber et al. 2012; Jain et al. 2017; Klein et al. 2022b; Linser et al. 2011a, 2014; Mandala et al. 2018; Retel et al. 2017; Schubeis et al. 2020; Söldner et al. 2023; Vasa et al. 2019; Zhou et al. 2007b) based on proton-proton distances. Apart from micro-crystalline proteins, this has been including various sample types from membrane proteins (Lalli et al. 2017; Retel et al. 2017; Schubeis et al. 2020; Shi et al. 2019; Zhou et al. 2012) and protein amyloids (Becker et al. 2023; Stanek et al. 2016; Xiang et al. 2017) to supramolecular assemblies (Lu et al. 2020; Zinke et al. 2020) and others. Moreover, complex magnetization transfer pathways, correlating multiple nuclei similarly to solution NMR, are becoming increasingly established (Ahlawat et al. 2023; Barbet-Massin et al. 2014; Fraga et al. 2017; Klein et al. 2022a, b; Linser et al. 2010b; Orton et al. 2020; Penzel et al. 2015; Sharma et al. 2020; Stanek et al. 2020; Vasa et al. 2018b). Important established experiments for resonance assignment so far are those solution-NMR-derived experiments correlating inter- and intraresidual carbon chemical shifts with amide ^15^ N/^1^H shifts similar to solution HNCA, HNCO, HNCOCA, HNCACO, HNCACB, and HNCOCACB (Akbey et al. 2009; Barbet-Massin et al. 2013, 2014; Knight et al. 2011; Linser et al. 2010b, 2011b; Zhou et al. 2007a). In these experiments, proton magnetization is utilized both for excitation and detection, and either scalar or dipolar transfers or mixtures thereof are used to connect the involved spins, with several possibilities of invoking relay spins dependent on the level/sites of protonation. As an extension to HNCACB correlations, sidechain-to-backbone correlations (“S2B experiments”) help to assess the sidechain carbon (Kulminskaya et al. 2015, 2016; Linser 2011) and/or proton chemical shifts (Ahlawat et al. 2023; Klein et al. 2022b; Stanek et al. 2016), thus providing residue-type information, which is key for mapping stretches of sequential correlations to the known primary sequence or enable H^ali^-based distance restraints for structure calculation (Andreas et al. 2016; Jain et al. 2017; Klein et al. 2022b; Nimerovsky et al. 2022; Söldner et al. 2023; Vasa et al. 2019). In addition to these H/N/C correlations, HNCACONH-type experiments, concatenating sequential amide groups directly, have been suggested (Andreas et al. 2015; Klein et al. 2022a; Orton et al. 2020; Xiang et al. 2015) as more effective sequential correlations that avoid ambiguities associated with the match-making process based on ^13^ C spins (“amide-to-amide experiments”).

For solid samples, transfer efficiencies are irrespective of the target’s molecular weight. Hence, for a given rotor diameter, preparation scheme, and experiment, the signal intensity obtained only scales linearly with the number of molecules in the rotor or inversely with the molecular weight of the target. (In the case of homooligomeric complexes or assemblies, this refers to the asymmetric unit.) Even though innovations for more complex sequences, but also for accelerated spectral acquisition (Gallo et al. 2019; Sharma et al. 2020; Stanek et al. 2020) and automated assignment (Klukowski et al. 2022; Lee et al. 2019; Schmidt and Güntert 2012; Volk et al. 2008) are soaring, manual, 3D carbon matchmaking-based assignment still seems to represent the established state-of-the-art. The assignment relies on the consolidation of multiple, usually at least five or six (backbone), possibly plus three (including sidechain assignments) experiments to fight ambiguities. Modern software packages, such as CCPNmr 3 (Skinner et al. 2016) or Poky (Lee et al. 2021), aid the user through the assignment process, but the assignment process inevitably becomes increasingly challenging with a growing number of spectra to consider even for an expert. A reduction of the number of spectra used while simultaneously reducing the number of overlapping peaks has been achieved by concatenating two 3Ds to a 4D experiment (Fraga et al. 2017; Klein et al. 2022b; Vasa et al. 2018b; Xiang et al. 2014; Zinke et al. 2017). This was shown to be advantageous both, for broad resonance lines (as in the cases of low deuteration levels or heterogeneous sample preparations) as well as for proteins with an increasing number of residues. The strategy is particularly beneficial if the number of magnetization transfers remains unaltered by the concatenation and losses in signal-to-noise derived from additional phase increments is exactly compensated by making the second experiment unnecessary, which is the case, e.g., in 4D (Vasa et al. 2018b) or 5D amide-to-amide experiments (Klein et al. 2022a; Orton et al. 2020).

**Fig. 1:**
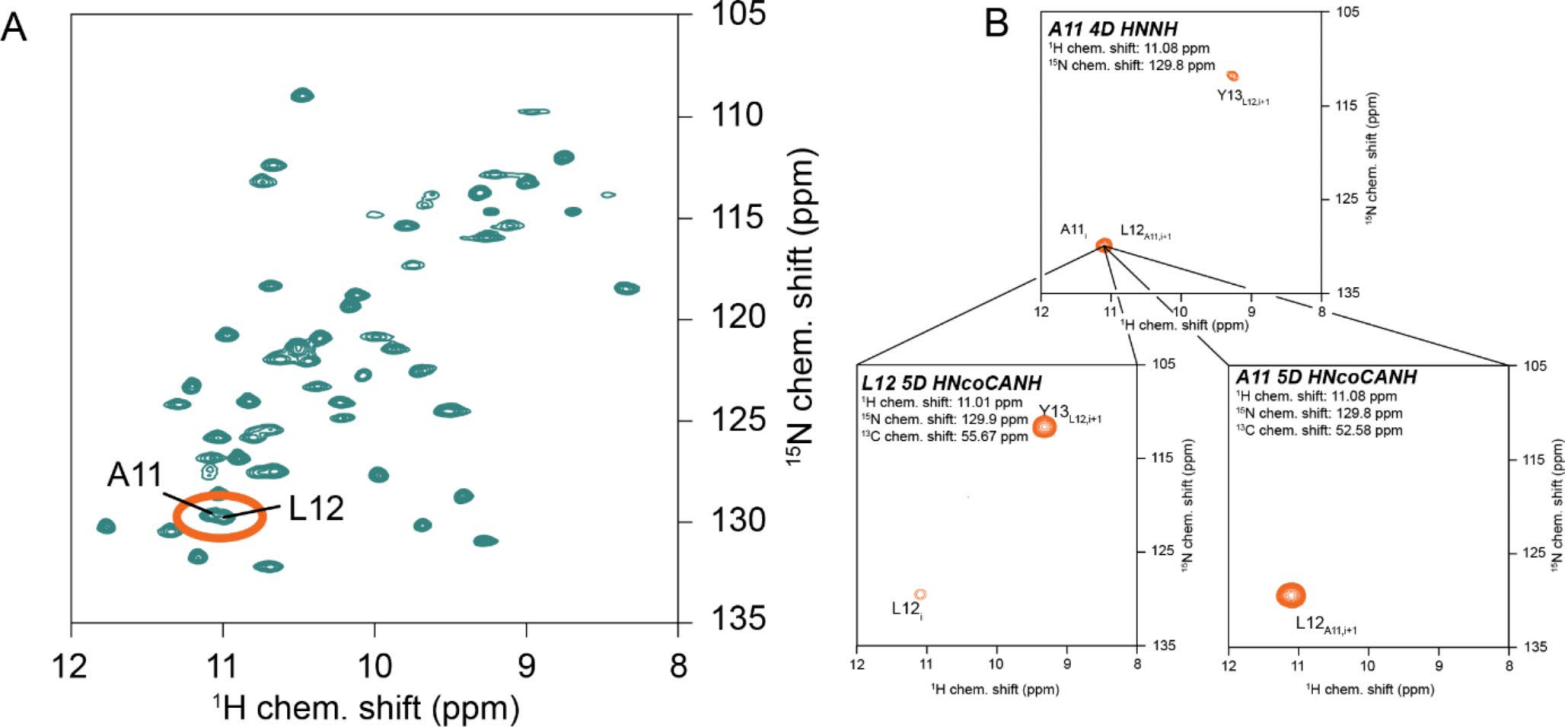
H/N overlap as found even for well-behaved targets. **(A)** 2D hNH spectrum of the SH3 domain of chicken α-spectrin. Despite its small size (62 residues) and high β-sheet content, the HN correlation comprises multiple overlapping peaks, causing ambiguities for any HN-based experiments, e.g., the standard out-and-back experiments. **(B)** Overlap in 4D HNNH direct amide-to-amide correlations (top), resolved by the additional carbon dimension in a 5D HNcoCANH experiment (bottom row), leaving only unambiguous assignments here

The pertinent hurdles of peak overlap and assignment ambiguity (the existence of multiple possible matches) of the process are excessively aggravated for an increasing number of residues, which entails an exponential growth of assignment possibilities. In the established approaches, a 2D H/X correlation (H/N or HA/CA) is used as a basis for identification of *i ± 1* resonances of the same kind. Even when the problematic step of carbon-based match-making is overcome by said direct sequential linkages suggested by us and Pintacuda et al. (Andreas et al. 2015; Klein et al. 2022a; Vasa et al. 2018b; Xiang et al. 2015), overlap of residue *i* in the source H/X plane (Fig. 1A) will lead to ambiguities in the assignment process, further amplified by overlapping peaks at the position of the *i + 1* signal and so on. 5D backbone experiments (Fig. 1) can partly levitate this limitation by a further increase of dispersion: In contrast to 4D HNNH amide-to-amide experiments, where only amide shifts are correlated with each other, for at least one of the two sequential amino acids they report on three nuclei, enabling more confident identification. This improves the concatenation of the sequential correlation with information from carbon-edited, in particular HNCACB-type and S2B-type, experiments, which allows for residue type information and hence for facilitated mapping of those correlations onto the primary sequence.

In previous work (Klein et al. 2022a, b) we have demonstrated that 5D data can be beneficial for backbone assignment of large protein complexes and sidechain proton assignments in fully protonated samples studied by solid-state NMR. One would assume that such efforts are only justified when “everything else fails”. However, here we would like to convince the reader that higher-dimensionality approaches offer competitive alternatives for a reliable and simple assignment even for the usual fast-MAS ssNMR targets of lower molecular weight, manually or in combination with automated assignment – which would commonly be addressed using lower-dimensionality strategies. Given the high transfer efficiency of proton-detected solid-state NMR spectroscopy, in particular, 5D HNcaCONH and 5D HNcoCANH amide-to-amide experiments are practically well feasible and can leverage a simplified, high-fidelity assignment process from a minimal set of experiments and can be easily combined with S2B experiments for sidechain assignments.

## Materials and methods

We used a perdeuterated sample of the SH3 domain of chicken α-spectrin, which was obtained by recombinant protein expression and purification in aqueous buffers as described previously (Linser et al. 2007). Micro-crystallization was achieved in aqueous buffer by pH-shift from 3.5 to 7.5, employing paramagnetic relaxation enhancement (PRE) with 75 mM Cu(EDTA) to accelerate data acquisition (Linser et al. 2007; Wickramasinghe et al. 2009). After precipitation overnight, the crystals were spun into a 1.3 mm rotor containing fluorinated rubber plugs, finally containing around 1 mg of protein. NMR experiments on the perdeuterated sample were carried out on a Bruker NEO spectrometer operating at a proton Larmor frequency of 700 MHz, using a triple-resonance HNC probe at 55.5 kHz MAS and approximately 25 °C. Detailed experimental conditions are listed in Tables S1 and S2. All 5D experiments were acquired using non-uniform sampling (NUS) with a Gaussian weighted, random schedule to minimize artifacts and interferences during reconstruction with the signal separating algorithm (SSA) as suggested by the authors (Kosiński et al. 2017). Exponential line broadening in the direct dimension was applied in all the spectra, while in some of the spectra, exponential apodization was applied in the indirect dimensions as well to maximize the signal-to-noise ratio. This ultimately results in a mixed window function along the indirect dimensions. 3D base experiments were recorded using either uniform or non-uniform sampling. NUS reconstruction was performed using SSA for 3D (Stanek and Koźmiński 2010) or 5D (Kosiński et al. 2017) experiments, respectively. Data analysis and manual assignments were done in CCPNmr V3 (Skinner et al. 2016), while FLYA (Schmidt and Güntert 2012) was used for automated resonance assignment. 5D experiments were recorded in blocks of 8 scans for ca. 2–2.5 days, after which a field reset, by tracking the shift of the water signal, was performed to account for the drift of the magnetic field in the absence of a lock signal. For the HNcoCANH experiment, three blocks of the same 2048 NUS points were recorded within a total time of ca. 6.5 days. The 5D HNcaCONH was acquired in two blocks, i.e., a total of 16 scans, and 1920 NUS points in a time of ca. 4 days. Addition of the individual blocks can be easily performed in TopSpin using *addser* as well as independent tools such as NMRPipe (Delaglio et al. 1995) or nmrglue (Helmus and Jaroniec 2013). The corresponding 3D base experiments were recorded as NUS experiments as well, using 16 scans and 1024 NUS points for the hCANH and the hCONH. The experimental time accounted for ca. 8 h in both cases. To obtain additional side-chain assignments, shifts from a 5D HCCNH were taken into account as recorded previously (Klein et al. 2022b). This 5D HCCNH experiment was recorded with a protonated sample on the same spectrometer using an HNC 0.7 mm probe at 100 kHz and approximately 20 °C. 

## Results

### Expansion of amide-to-amide experiments to 5D

**Fig. 2:**
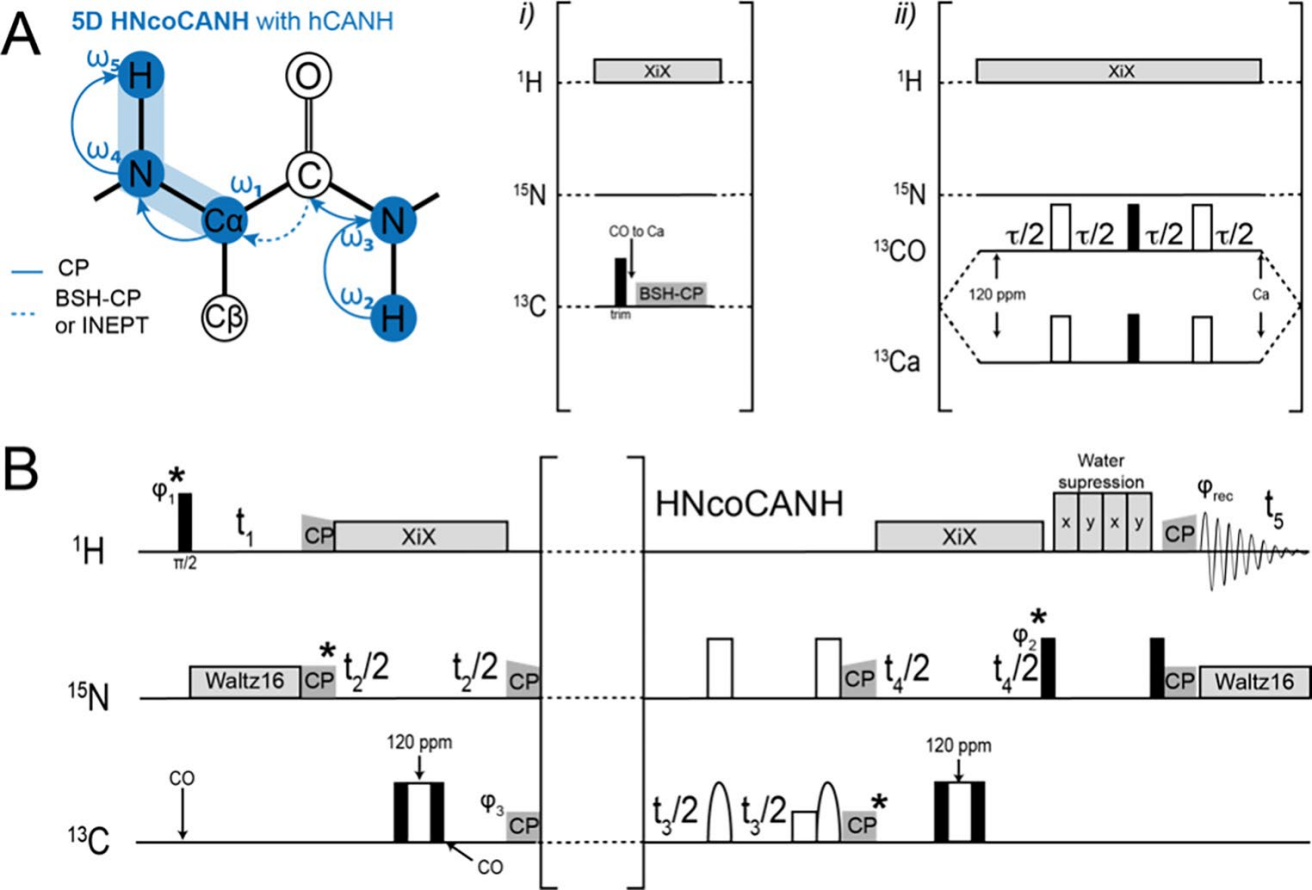
5D magnetization transfer scheme and pulse sequence for the HNcoCANH using carbon-carbon CP (i) or INEPT (ii). Both versions have been used previously (Andreas et al. 2015; Klein et al. 2022a; Orton et al. 2020; Stanek et al. 2020; Xiang et al. 2015) but are shown here again for completeness. For the INEPT transfer step, using a block similar to the one reported in Barbet-Massin et al. 2013 (Barbet-Massin et al. 2013), only hard pulses at a ^13^ C carrier position of 120 ppm were used here. Compare Orton et al., also involving selective pulses (Orton et al. 2020). Phase cycling for HNcoCANH: φ_1_ = x,-x; φ_2_ = x,x,-x,-x; φ_3_ = x,x,x,x,-x,-x,-x,-x; φ_rec_ = x,-x,-x,x,-x,x,x,-x. Carrier changes are marked by arrows. The Cα carrier position was set to 55 ppm, while 173 ppm was used for carbonyl carbons. Water suppression was achieved by the MISSISSIPPI scheme (Zhou and Rienstra 2008) without gradients, using a pulse length of 20 ms at 10 kHz rf field strength

The pulse sequences for 5D HNcoCANH and 5D HNcaCONH (Fig. 2) here are obtained straightforwardly from their lower-dimensionality (HNNH) variants (Andreas et al. 2015; Xiang et al. 2015) and feature an additional carbon indirect dimension *t*_1_. Both 5D experiments were introduced as NUS or APSY previously (Klein et al. 2022a; Orton et al. 2020) using exclusively CP for heteronuclear transfers and either BSH-CP (Chevelkov et al. 2013; Shi et al. 2014) or INEPTs for homonuclear CC transfer. The two sequences are composed of the same number of transfer steps as their lower-dimensional counterparts. In the case of an INEPT transfer (Fig. 2B C), starting with in-phase CO magnetization, only the CO-Cα coupling is active during the INEPT transfer, which allows simple rectangular pulses. Even though during the refocusing period, the Cα-Cβ coupling is also active, hard pulses were again used here to avoid the complication of poor separation of Cα and Cβ resonances for Cα-selective pulses and losses during the rather lengthy selective pulses (Barbet-Massin et al. 2013).

**Fig. 3:**
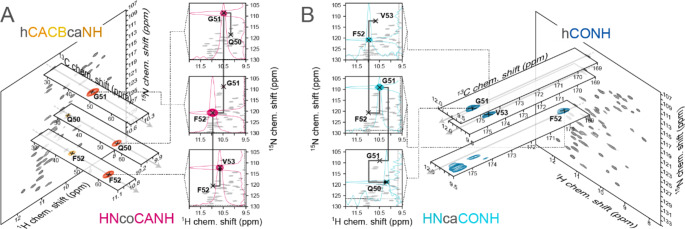
5D amide-to-amide backbone walks, illustrated for the HNcoCANH **(A)** and the HNcaCONH **(B)** experiment. Both experiments enable a straightforward backbone walk, as they provide direct, well-resolved *i* to *i* + 1 connectivities. If sidechain carbon information is desired anyways, an hcaCBcaNH with split CACB transfer can be used instead of an hCANH as the base experiment for 5D reconstruction. The HNcaCONH benefits from the higher resolution of the carbonyl carbons but lacks the possibility of connecting sequential with sidechain information

In the following, using the SH3 domain of chicken α-spectrin, we demonstrate that the above strategies are a competitive approach for a virtually complete, highly effective, and user-friendly assignment process on small proteins that have been tackled with standard methodology. To identify a minimal set of higher-dimensional experiments required, we recorded different 5D backbone experiments on a perdeuterated and 100% proton back-exchanged sample and expanded the set by a sidechain-to-backbone (S2B) experiment acquired on a fully protonated, micro-crystalline sample. Figure 3 shows an exemplary set of 2D slices from the 5D HNcoCANH experiment (Fig. 3A) as well as from the reverse HNcaCONH experiment (Fig. 3B) in combination with a suitable 3D base experiment each. When Cβ information is sought, a 3D hcaCBCANH (using 1/(4* J*)) experiment is often competitive to the 3D hcaCBcaNH with full *J*-transfer (over 1/(2* J*)). This experiment includes both, Cα and Cβ shift information and can equally be used as a base experiment for reconstruction of the 5D directly instead of a 3D hCANH, which then becomes dispensable.

### Data processing and spectral features

**Fig. 4:**
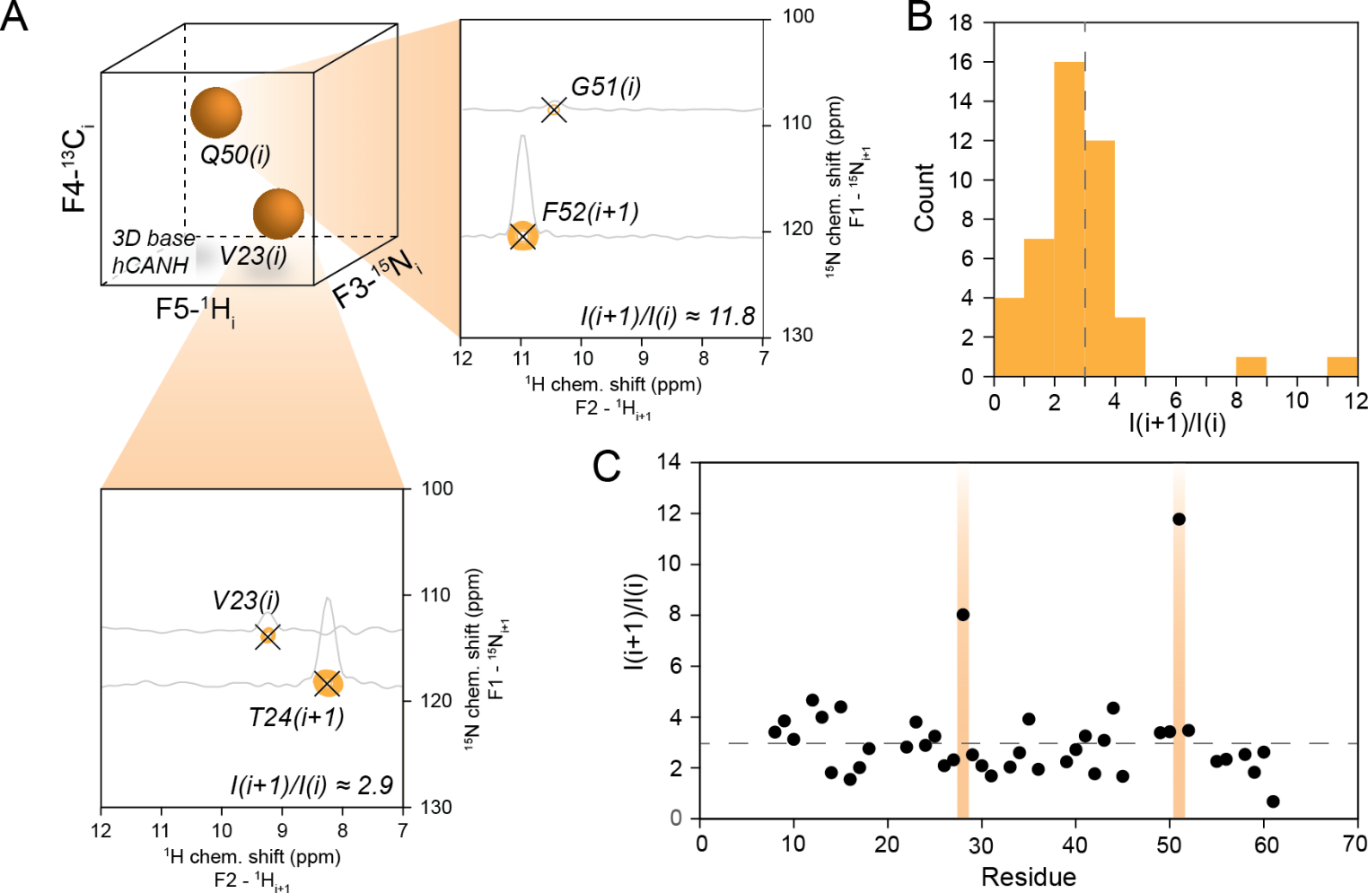
Processing of 5D HNcoCANH experiment and information obtained. **A)** Using SSA for spectral reconstruction, a stack of 2D planes is returned (one for each previously defined 3D peak). **B and C)** In BSH-CP versions of the amide-to-amide correlations, incomplete magnetization transfers cause additional out-and-back-like correlations with a constant intensity ratio of around 3:1 for non-glycine residues but much higher for glycines (shown as a histogram in **B)** and as a function of sequence in the SH3 domain in **C)**

Recording NUS 5D experiments is straightforward using standard software. Processing of 5D data sets, on the other hand, can be leveraged by the SSA algorithm using sparse multi-dimensional Fourier transformation (SMFT), developed by Koźmiński and coworkers (Kazimierczuk et al. 2009; Kosiński et al. 2017; Stanek and Koźmiński 2010). In brief, this methodology does not reconstruct the entire 5D frequency domain spectrum but instead produces frequency-domain data only at predefined positions. These regions are preselected through a peak list obtained from picking a 3D spectrum that shares three dimensions with the 5D experiment (see Fig. 4A). The approach avoids the excessive amount of data and computation time required if 5D data sets were fully Fourier-transformed, using artifact reduction in all indirect dimensions at defined regions of the 5D space (Kosiński et al. 2017; Stanek and Koźmiński 2010). As undefined peaks, i.e., not peak-picked ones, will not be processed by SSA, high signal-to-noise ratio and resolution are desired in the 3D base experiments, which can again be leveraged via NUS. The experiments proposed in this work for the 3D base experiment are the 3D hCANH or 3D hCONH experiments, rather than the alternative hCAcoNH or hCOcaNH experiments, due to their better sensitivity.

The 5D experiments share an H/N/C shift triplet with the base experiment. Therefore, only the additional H and N dimensions, providing chemical shifts of the *i + 1* residue (HNcoCANH) or the *i-1* residue (HNcaCONH), are generated for each 3D source peak provided. The processed 5D spectrum eventually becomes a stack of 2D H/N planes at predefined H/N/C peak positions that can be superimposed onto ordinary 2D H/N correlation spectra. Evaluation can be done via common software packages, like NMRFAM-SPARKY (Lee et al. 2014), POKY (Lee et al. 2021), CCPNmr V3 (Skinner et al. 2016) etc. and does not require special data processing other than NUS reconstruction to make the spectra accessible. In addition to the gain in resolution, the absence of depth through a third dimension in 5D spectra processed this way strongly reduces the level of complexity upon analysis. In particular, the difficulty of finding and evaluating peaks and determining their maxima in three or four dimensions is replaced by mere 2D peak identification.

Selecting peak *i* of a sequential correlation via *three* coordinates effectively avoids peak overlap, which not only occurs for complex proteins or heterogeneous samples but even for small, micro-crystalline proteins (compare Fig. 1), and therefore facilitates identification of next- (or previous-) residue H/N shifts with high fidelity compared to HNNH 4Ds in a general sense. Not only the sparsity of peaks in 5D space, largely reducing overlapping correlations, and the direct residue linking using amide shifts dispensing the drawbacks of carbon-based matchmaking, but also the facilitated concatenation of sequential-walk information with residue-type information from hcaCBcaNH or S2B experiments (linked to the sequential correlations via the H/N/Cα triplet) are general advantages of the approach applicable for a wide variety of protein targets.

The *i + 1* (HNcoCANH) or *i-1* (HNcaCONH) peak, respectively, is the predominant signal found in the spectra and easily distinguished from remaining NUS artifacts or thermal noise in the regime of conditions described here. Interestingly, in the BSH-CP version, due to the possibility of magnetization transfer in CP steps to nuclei two bonds away, an out-and-back transfer to the H/N group of origin can be observed, leading to an additional (“diagonal”) peak in the processed 2D plane. This out-and-back pathway can likely be attributed to the upper-limit magnetization transfer of around 50% during the BSH-CP (Chevelkov et al. 2013; Shi et al. 2014). As similar CP conditions apply for NCA and NCO transfers, non-transferred CO magnetization after the BSH-CP may partially be transferred back to the amide nitrogen of origin. Whereas this diagonal peak is redundant and has to be taken into account upon judgement of bulk signal intensities (see below), it in fact practically facilitates identification/confirmation of the source resonances upon manual inspection. Rigorous analysis of the HNcoCANH spectrum shows that the ratio between the *i + 1* and *i* peak (cross peak to diagonal peak) is constant at approximately 3:1 for all amino acids apart from glycine (Fig. 4B/C) which show a ratio of ca. 8:1. We ascribe these effects, which might be exploited for residue type identification, to higher BSH-CP transfer efficiency in the case that no Cβ is present (The latter may function as a sink of magnetization for *rf* fields close to or at the HORROR condition.) as well as possibly the NCO and NCA conditions being somewhat more distinct for Glycines.

### Sensitivity considerations

**Fig. 5:**
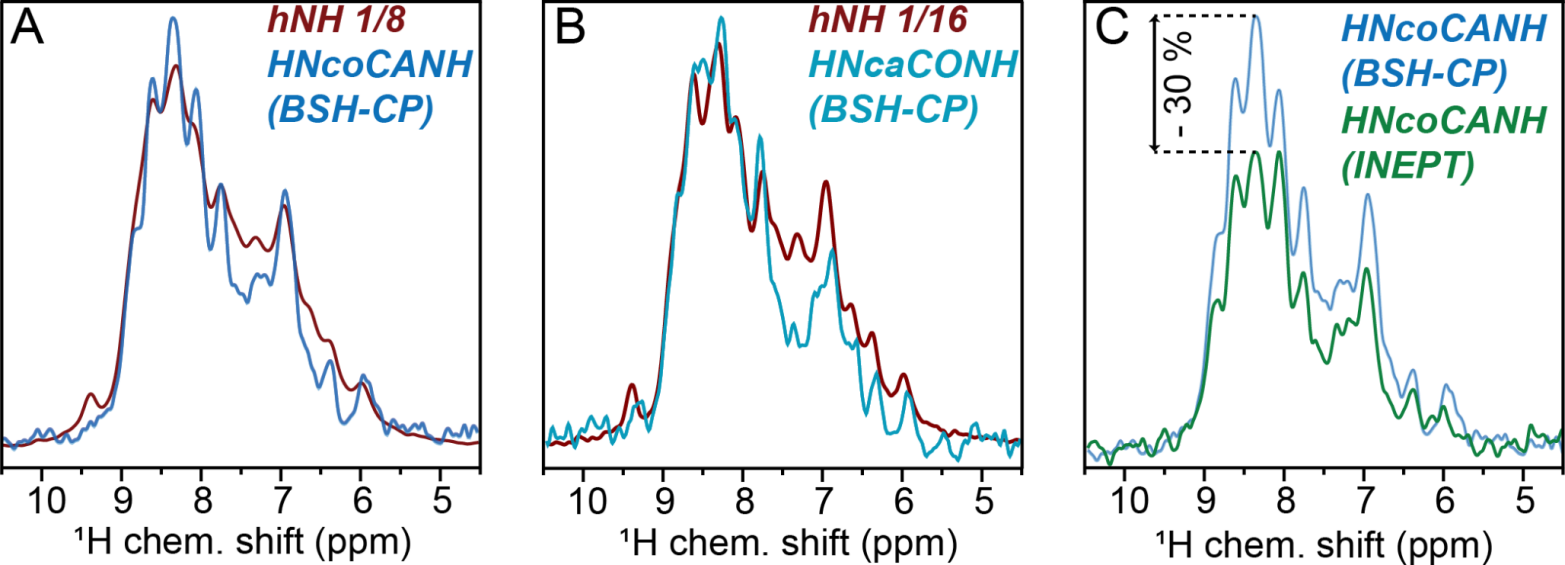
Bulk signal intensities for the 5D HNcoCANH and 5D HNcaCONH compared to the hNH intensity. **(A)** The HNcoCANH maintains approximately 12% of the hNH when used with BSH-CP for homonuclear CC transfers. **(B)** Using the HNcaCONH with BSH-CP results in ca. 6% of the hNH bulk signal. **(C)** In case an INEPT block is used, the signal intensity is attenuated by an additional 30%, however, in contrast to the BSH version, the bulk signal represents only the peaks of interest. Also, the difference between CACO and COCA is not observed in the INEPT version

Figure 5 shows a comparison of bulk signal intensities for the same sample. HN bulk intensities of 12% and 6% are obtained in case of the HNcoCANH and the HNcaCONH, respectively, which is within the range of expected performance (Penzel et al. 2015). A strong difference between the CO-Cα and the Cα-CO BSH-CP is noted, which is in line with previous quantification of this pulse sequence element using DREAM transfer (Penzel et al. 2015) and presumably derives from competing Cα-Cβ transfer. For the perdeuterated and amide back-exchanged protein in a 1.3 mm rotor, the INEPT-based scheme (element *ii)* in Fig. 2) yields slightly reduced intensity of the HN bulk signal compared to the fully CP-based HNcoCANH version in our hands (Fig. 5C). However, almost all of this signal can be assumed to reflect the signal of interest, whereas in the dipolar sequence a quarter of the overall signal derives from back transfer (the diagonal peak, see above), making the INEPT version similarly sensitive in practice. Here, no diagonal peaks (no back-transfer) are/is evident as tested in 3D NNH-type experiments (hNcocaNH, recorded on a fully protonated sample), see Fig. S1. Given that for INEPTs, in contrast to BSH-CP, the Cα-CO transfer (in the other direction) performs with similar efficiency (Andreas et al. 2015; Penzel et al. 2015; Xiang et al. 2015) as the CO-Cα transfer, the INEPT version is a generally competitive alternative whenever heteronuclear *T*_2_ times are sufficiently long (i.e., using deuterated samples or MAS of around 100 kHz or faster).

Naively, the increased number of transfer steps in the direct amide-to-amide experiments would suggest extremely poor sensitivity compared to assignment strategies based on complementary carbon matchmaking. In addition to the advantages in assignment fidelity, however, the sensitivity of the approach is in fact very competitive, as the set consisting of carbon matchmaking experiments relies on a greater number of individual experiments, including some with very low sensitivity: hCANH and hCONH schemes provide a bulk signal to noise of around 25%, however, their counterparts (hCAcoNH and hCOcaNH) only bear on the order of around 12%, due to losses during the carbon-carbon transfer step (Penzel et al. 2015; Shi et al. 2014). The pair of hcaCBcaNH and hcaCBcacoNH experiments only provide a bulk signal of around 8 and 3%, respectively. Even if, hypothetically, the full suite of six carbon-based 3D experiments did carry the same fidelity for unambiguous matchmaking as a 5D amide-to-amide correlation (which is not the case as explained above) and if an additional hcaCBcaNH is always desired even as a supplement for the latter case, the measurement time requirements would be higher for the set based on carbon-matchmaking-based 3Ds (see Table 1). (In this direct comparison with 3D experiments, an assumed 10% bulk signal of the 5D amide-to-amide correlation would have to be corrected by a √2 for each additional indirect dimension compared to a 3D (i.e., a factor of 2) as well as the ratio between intensity of the peak of interest and overall magnetization transfer derived from the bulk signal (0.75). This number is still higher than the bulk sensitivity of the weakest one (the hcaCBcacoNH, ca. 3%) of the six 3D experiments. It is further important to note that NUS spectra generally show equal or higher signal-to-noise ratios compared to time-equivalent US spectra when sampled with a decaying sampling function, e.g., exponential or gaussian weighting (Hyberts et al. 2013; Palmer et al. 2015; Paramasivam et al. 2012), the more indirect dimensions are present. 5D sensitivities therefore scale up favorably towards improved sensitivities. The actual scaling factor, however, is strongly depended on the bias of the sampling function as well as the recorded *t*_max_ in each indirect dimension, and 1.2-fold – 3-fold improvements per indirect dimension can be achieved (Palmer et al. 2015; Paramasivam et al. 2012; Suiter et al. 2014). For 5D and 4D experiments, the many indirect dimensions therefore tend to lead to substantial sensitivity gains. (To take this effect into account in Table 1, we also listed tentative numbers (in brackets) assuming an improvement of 1.2x per indirect dimension. (As also 3Ds can of course be recorded as NUS experiments, such number are also shown for those – again in brackets.) In addition to easing the biggest bottleneck of the set, *fewer* experiments have to be recorded for the 5D set, streamlining the actual assignment process for the user (see below for assignment success with different kinds of experiments taken into account within a computational framework). Even whenever hcaCBcaNH and hCONH are desired in any case (e.g., for complementing the 5D approach for residue-type information and secondary-structural analysis) and the hCANH is additionally recorded to serve as a basis for SMFT (As mentioned above, the hcaCBCANH can be used for this purpose, too.), the 3D hCAcoNH and 3D hCOcaNH become dispensable. The advantage of several-fold time saving stays even when the six 3Ds are all recorded under NUS (assumed 1.2x intensity gain per indirect dimension, numbers in brackets) and even when sampling-related sensitivity gain in NUS is ignored altogether, even for the 5D (numbers in squared brackets).

Even when the most insensitive experiment of the traditional set (the hcaCBcacoNH) is omitted (Set 2,3,4,6,7, with a relative time to 2D hNH of 710 – which can work for very well-behaved proteins as the SH3 domain in the following but further reduces the fidelity of sequential connections to the dispersion of the CO/Cα 2D plane), the time required for the 5D set with its direct connections (relative time to 2D hNH also being 710) is still on par. With the possibility of sensitivity enhancement for all or most of the indirect dimensions (Blahut et al. 2022), further strong increase of the competitiveness of higher-dimensionality approaches vs. standard approaches can be expected.

**Table 1:** Comparative measurement times required for different experiments/sets based on the amide bulk signal and dimensionality-based (phase-sensitive incrementation) corrections, relative to a 2D hNH of the same sample under the same conditions. Numbers in brackets denote expected changes for also recording the 3D experiments using NUS. Squared brackets denote estimates where NUS-related sensitivity gains are fully neglected. The factor 0.75 in the 5D takes into account that the peak of interest only makes up ¾ of what is observed in the bulk signal, whereas ¼ is the redundant “diagonal” peak (see below)

**No**	**Exp.**	**Sens. rel. to 2D hNH**	**Time to hNH**	
1	5D HNcoCANH	10%/2^3/2^*1.2^4^*0.75	330 [1400]	**Set 1, 2, 3, 4:**
2	3D hCANH US (*NUS)*	25%/2^1/2^ (*1.2^*2*^)	32 (15)	710 (510) [1800]
3	3D hcaCBcaNH US (*NUS)*	8%/2^1/2^ (*1.2^*2*^)	310 (150)	**Set 2–7:**
4	3D hCONH US (*NUS)*	25%/2^1/2^ (*1.2^*2*^)	32 (15)	2900 (1400)
5	3D hcaCBcacoNH US (*NUS)*	3%/2^1/2^ (*1.2^*2*^)	2200 (1100)	**Time saving:**
6	3D hCAcoNH US (*NUS)*	11%/2^1/2^ (*1.2^*2*^)	170 (80)	4.1 (2.8) [1.6]
7	3D hCOcaNH US (*NUS)*	11%/2^1/2^ (*1.2^*2*^)	170 (80)	

A direct, purely experimental signal-to-noise comparison for a given peak would be preferred over the above theoretical assessment but is compromised by the necessity of NUS (and reconstruction) for the 5Ds and the associated difficulties of measuring noise there. (The acquisition of four indirect dimensions in a uniform manner is practically infeasible.) However, the propagation from bulk signal-to-noise is straightforward and rigorous when considering all relevant factors. In either way, one has to keep a sample- and setup-dependent variability of transfer efficiencies and NUS reconstruction in mind.

## Application to the SH3 domain of chicken α-spectrin

In a *manual* backbone walk based on the 5D HNcoCANH experiment recorded on the SH3 domain with 8 scans and 1805 NUS points (36 h on a 700 MHz spectrometer), 46 out of 48 expected resonances can be assigned. Concatenated with a 3D hcaCBCANH (2d), complete backbone assignment can be obtained from this minimal set of experiments. In order to see whether assignments improve with an increased signal to noise ratio, we recorded the HNcoCANH step-wise longer in blocks of 2 days and with slightly more NUS points (up to in total three blocks of 8 scans, 2048 points) and again performed a manual assignment. Naturally, the best spectrum was obtained after the longest (6 days of) measurement time, but the two weakest resonances were unambiguously reconstructed after 4 days (or two blocks). Assuming experimental times of approx. 2 days for a uniformly sampled 3D hcaCBcaNH and 12 h for an hCONH experiment (compare Table 1), a complete backbone assignment can therefore be realized within 6.5 days. This time is on par or faster compared to established 3D-based strategies, but warrants reduced ambiguity and a more intuitive and straightforward assignment procedure. Alternatively, the backbone walk can also be followed using the HNcaCONH experiment. However, when recorded with 16 scans and 1920 NUS points, only 41 out of 48 expected resonances can be unambiguously assigned.

**Fig. 6:**
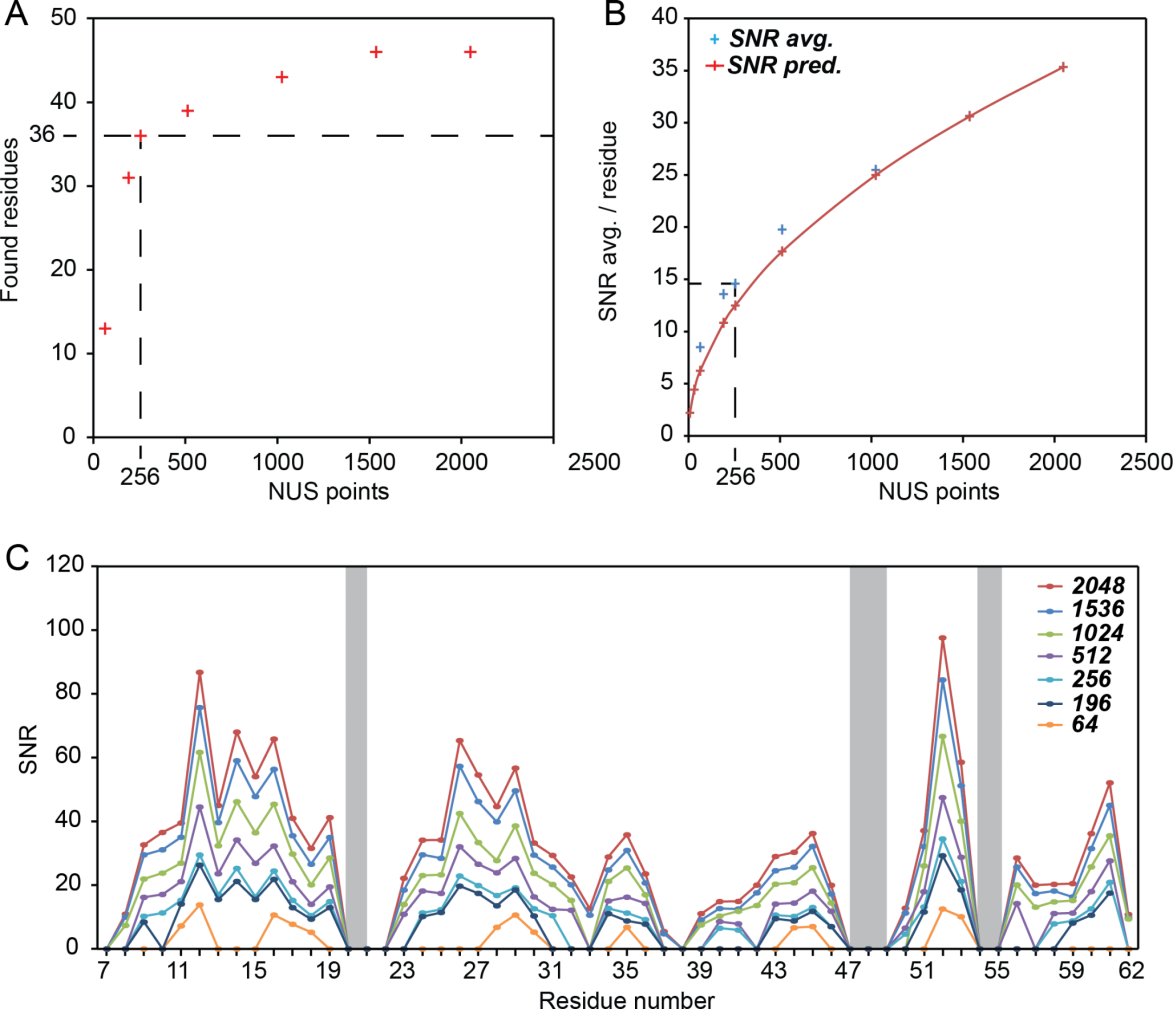
Influence of the number of NUS points (complex time points) and hence overall experimental time invested on the number of signals, i.e., residues found in the spectrum (**A/C**) and the signal-to-noise ratio (SNR) (**B/C**) after reconstruction of the FID recorded with 8 scans (minimal phase cycle). The dashed lines in (**A**) mark the threshold at which 75% of all 48 possible signals are found, arbitrarily chosen as the limit for a minimal acceptable performance. In (**B**) the averaged SNR over all identified signals is shown with blue crosses, while the red curve marks the calculated SNR extrapolated down from the SNR measured for 2048 NUS points. The dashed lines mark the SNR reached for the 256 points, for which 75% (36 out of 48) of all signals are found. In (**C**) the signal-to-noise ratio for the individual residues is shown in dependence of the number of NUS points included for reconstruction. Prolines 21 and 54 and CP inaccessible residues 47 and 48 are marked in gray (problematic both in the roles of residue *i* or *i*-1). R21 and N38 are mobile residue always difficult to detect. (Linser et al. 2010a) The data reduction (i.e., reduction of measurement time effectively included in the sparse FID) was implemented by truncating to only the desired subset of time-domain points. As the NUS schedule was randomized before acquisition, the maximum resolution and sampling density weighting is maintained for all subsets. 2048, 1536, 1024, 512, 256, 192, and 64 time-domain complex points correspond to effective experimental times of 52 h, 39 h, 26 h, 13 h, 6.5 h, 5 h, and 1.5 h, respectively

Maybe more interestingly, to assess the minimal required measurement time for a given performance, we artificially truncated the sparse FID recorded with a minimal phase cycle of 8 scans (2048 points, 52 h experimental time), leaving either 1536, 1024, 512, 256, 192, or only 64 time-domain complex points (Fig. 6), which is commensurate to 39 h, 26 h, 13 h, 6.5 h, 5 h, and 1.5 h experimental time, respectively (Fig. 6A). As the signal-to-artifact ratio improves with the square root of recorded NUS points (Kazimierczuk et al. 2009) and the thermal signal-to-noise ratio (SNR) generally improves with an increasing number of FIDs (assuming t_max_ < 1.3 T_2_), maximizing the number of NUS points over the number of scans in a time-equivalent manner will usually lead to equivalent or better spectra. In case of the SH3 domain, > 75%, i.e., 36 out of 48 possible resonances, can be identified starting from around 256 NUS points with 8 scans (i.e., around 6 h of measurement time). For this condition, an average SNR of ca. 15 is achieved, whereas for shorter times the spectral quality breaks down (Fig. 6). Considering the quadratic dependency of the SNR and the molecular weight, i.e., copies of the molecule in the rotor, a protein of 35 kDa or 5-times the size of the SH3 domain would require at least 25-times more scans, i.e., 6400 NUS points (when the minimal phase cycle of 8 is employed). It should, however, be emphasized here that this is merely a qualitative approximation as spectral crowding may require an even higher number of NUS points. Besides the molecular weight, the SNR of (any) NMR experiment heavily depends on other factors, such as sample packing, homogeneity, etc. and the above recommendations will only be partially translatable, which in turn would require constant processing and evaluation of the 5D until convergence is reached. To introduce another qualitative guideline, the SNR of an hCANH experiment recorded for the same sample over 12 h (the one that we used as a source spectrum for the SMFT reconstruction of the 5D HNcoCANH) is depicted as a function of residue in Fig. S2. This comparison might be used to qualitatively estimate the required measurement time for a 5D to reach the different levels of performance shown in Fig. 6 for other samples that an hCANH has previously been recorded for. Here, the hCANH signals show a SNR of around 20–40 after 12 h of measurement time with similar trends for the individual residues as the 5D (Fig. S2). If for future samples the 3D will take *n*-times longer to reach a similar average SNR, the measurement times, i.e., minimum number of NUS points, for the 5D would accordingly have to be increased by *n*. (Mind the general inaccuracies upon quantifying the SNR of NUS data sets.)

**Fig. 7:**
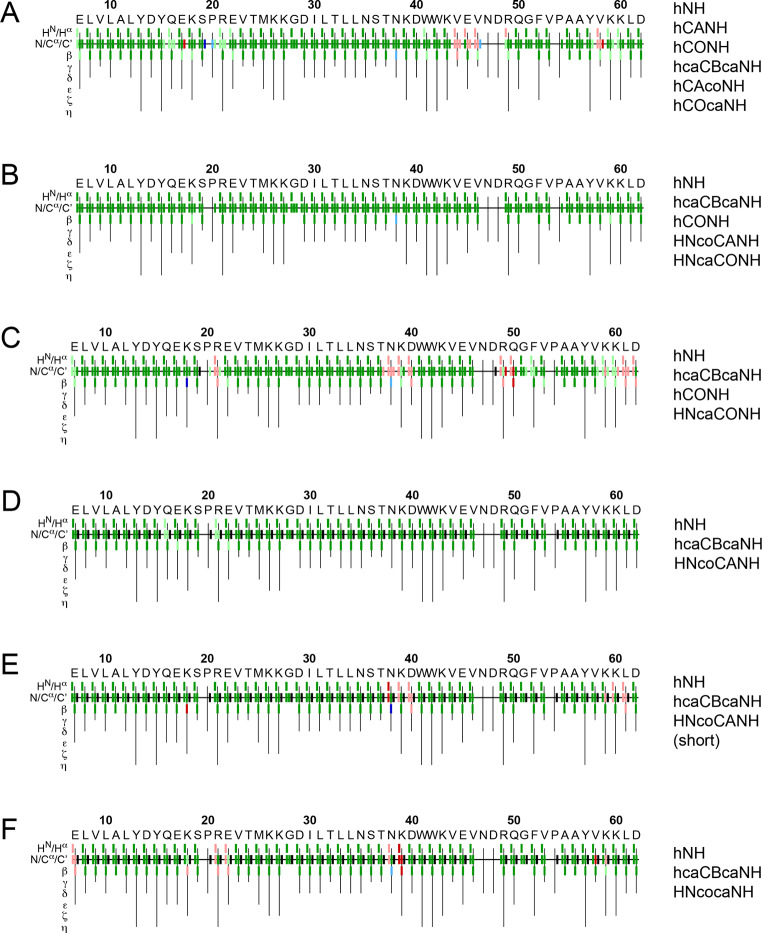
FLYA assignments using different levels of input data. The classical set of 3D experiments for backbone assignment was used as input for FLYA in **(A)** and can be seen as a point of orientation for the performance of FLYA. For **(B)** to **E)**, different combinations of 5D and 3D experiments were used (see the text for more details, HNcoCANH with 24 scans in B) and (C) and with 8 scans in E). **F)** The 5D HNcoCANH was replaced with a 4D HNcocaNH experiment, yielding slightly worse but similar results. For all runs, the manual assignments were used as a reference. 100 independent runs were performed, using tolerances of 0.1 ppm for ^1^ H and 0.5 ppm for ^13^ C and ^15^ N, respectively, and ultimately combined into a consensus chemical shift assignment. (A 2D hNH is usually submitted as well, but implies negligible costs, relatively speaking.) Color scheme: Green: In agreement with reference assignment (here the manual assignment). Red: Disagreement with reference assignment. Blue: Additional assignments, not in reference. Black: In reference but not assigned by FLYA. Dark colors generally indicate “strong”/reliable assignments that are found in at least 80% of the independent FLYA runs, lighter colors indicate they have been found less than 80% of the time

*Automated* assignment approaches, which have been becoming increasingly powerful throughout the last years, are becoming an indispensable support for leveraging resonance assignment of large proteins. Nevertheless, they also streamline the assignment of smaller proteins to the point of fully automated peak picking, assignment, and structure calculation (Klukowski et al. 2022) and can be generally used as a tool to validate manual assignments. Figure 7 shows the application of the FLYA algorithm (Schmidt and Güntert 2012) for the peak list obtained from different combinations of 5D experiments and different other experiments for automated assignment of the deuterated sample of the SH3 domain. The caption of Fig. 7 explains the commonly used color coding of FLYA, which reports (dis-)agreement with reference assignments as well as reliability of the automated assignments. These results are validated by comparison with the manual assignments obtained from 3D experiments for the residues visible in CP-based experiments, i.e., excluding residues 1–6 and 47–48. (These residues escape CP-based schemes due to their extensive fast dynamics.) The classical set of 3D experiments, however leaving out the hcaCBcacoNH as the weakest and most time-consuming one yields 99% strong assignments that are correct (Fig. 7A; Table 2). (For the SH3 domain no carbon-matchmaking ambiguities are present in the Cα/CO 2D plane, hence retaining hCANH, hCAcoNH, hCONH, hCOcaNH, and hcaCBcaNH is sufficient. In other words, for this set of five 3D experiments FLYA is in agreement with the manual assignments; exceptions are residues 44–46 at the beginning of the distal loop. A similar outcome is obtained for each of the combinations including 5D amide-to-amide correlations (Fig. 7B-F; Table 2). The weaker HNcaCONH suffers from an overall reduced number of visible peaks and reduced chemical shift redundancy (Fig. 7C; Table 2). The combination of both 5Ds and their base experiments, on the other hand (here, the hcaCBCANH was always used as a base), returns complete and 100% correct assignments (Fig. 7B; Table 1) but requires increased measurement time investments. However, nearly perfect assignment is obtained also for a minimal set of one (HNcoCANH, here with 24 scans) 5D amide-to-amide experiment in conjunction with the hcaCBCANH (Fig. 7D; Table 2, no errors). When the short 5D HNcoCANH is employed (i.e., the 36 h version), FLYA yields remarkably complete and correct assignments (Fig. 7E; Tables 2 and 98% correct, i.e., 2% mismatch, in total approx. 3.5 days of measurement time). Only two resonances are erroneous, which are next to two residues that are not always correctly assigned by FLYA. Interestingly, the two residues directly missing in the 5D (R21 and T37), can be indirectly assigned by FLYA, probably in the same way the expert is able to assign those in the manual assignment process. Importantly, in both of these cases (Fig. 7D and E, and Table 2), backbone assignments are obtained from only *two* experiments, avoiding lots of the error-prone peak-picking and minimizing combinatorial efforts of standard assignment tasks. (A reduced set of experiments also simplifies chemical-shift referencing, a common problem for solid-state NMR as a lock signal is absent.) For comparison, a FLYA run using a 4D HNNH instead of the 5D HNcoCANH yields similar results to the run with the quick (36 h, lower-s/n) 5D (Fig. 7F; Tables 2 and 97% correct, 3% mismatch). This 4D spectrum was recorded for approx. 30 h (581 NUS points and 16 scans), i.e., it would have a higher signal to noise than the combination in Fig. 7D. As such, as expected, the vacancies in the assignment are a result of H/N overlap rather than signal to noise. Missing CO resonances can in either case be filled in a straightforward way either by FLYA (see Fig. S3, no strong violations after 36 h) or the user. The other residues missed out even for excessive sets of experiments (Fig. 7B/D and Table 2) are invisible in any CP-based experiments and have been assigned previously only via fully INEPT-based experiments (Linser et al. 2010a).

**Table 2:** Completeness and correctness of the individual FLYA runs depicted in Fig. 7 above. The naming of the runs follows the elements in Fig. 7. The last column indicates all assignments made by FLYA irrespective of their reliability and correctness, i.e., also “weak” assignments and erroneous assignments are considered. The first three runs included the hCONH experiment and therefore have a higher number of overall assignments, whereas in runs 7C, 7D, and 7F no CO shifts are provided and can hence not be assigned. The second column from the right considers only the “strong” assignments but does count erroneous ones, in contrast to the third from the right, which only counts the “strong” assignments that agree with the reference

Run	Correct and strong	Overall strong	All assigned
7A	208	211	259
7B	253	253	256
7C	210	219	256
7D	197	197	205
7E	189	193	206
7F	183	188	206

## Simultaneous backbone and sidechain assignment

**Fig. 8:**
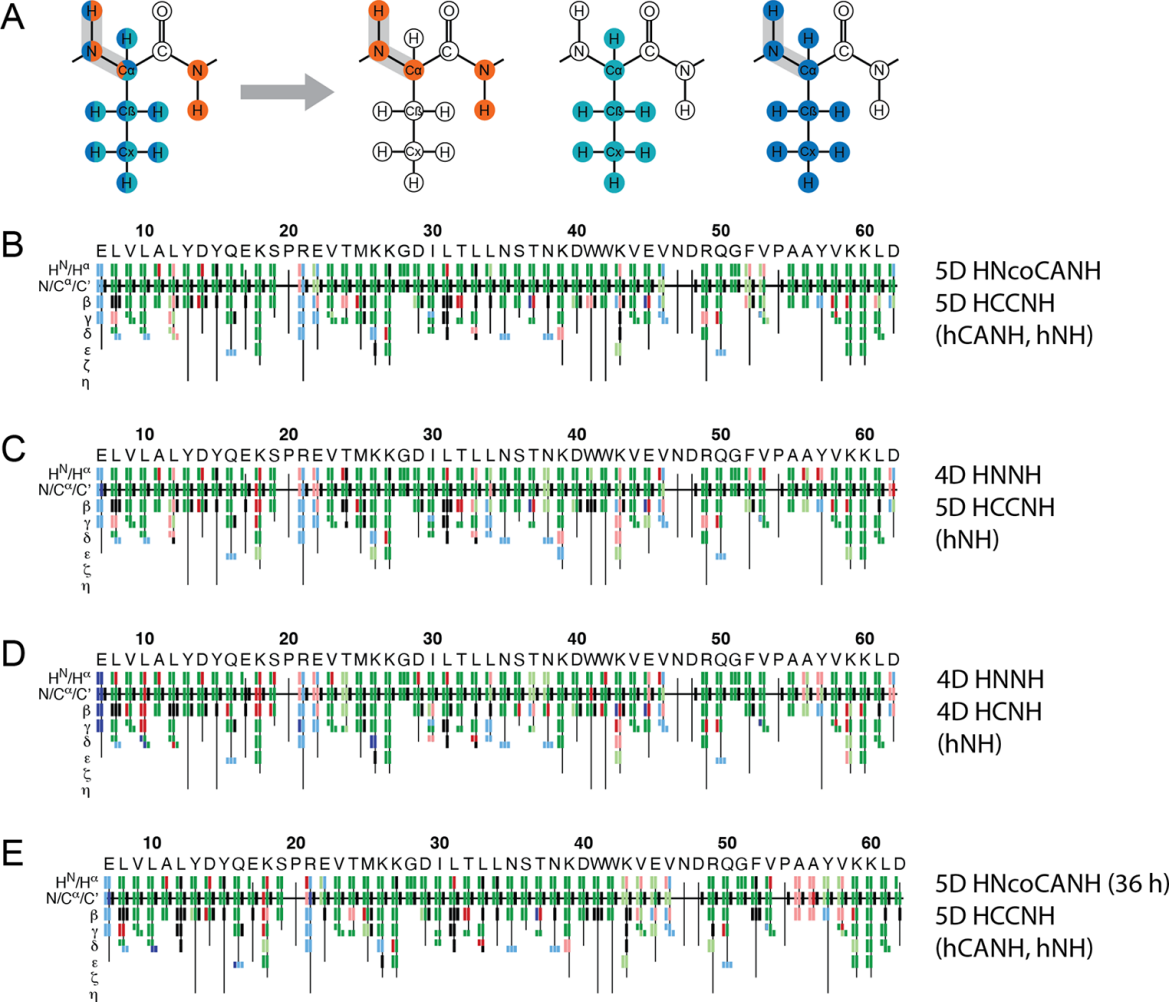
Results of FLYA assignments via an experimental pair of an amide-to-amide experiment and an S2B experiment in comparison to the manual assignments. **(A)** Sketch of magnetization transfers of the experiments in the set. The orange HNcoCANH was recorded on a 1.3 mm sample, the 4D HCCH (cyan) is an additional pathway available for free while recording the 5D HCCNH (blue); both were simultaneously recorded on a 0.7 mm sample. **(B)** to **E)** FLYA results from different combinations of input data as denoted on the right. Although the assignment success is very similar for the different combinations, the best agreement with the manual assignments is achieved through the combination of two 5Ds, likely due to the higher chemical shift redundancy. The tolerances used are 0.1 ppm for ^1^ H, 0.8 ppm for ^13^ C, and 0.5 ppm for ^15^ N, respectively. Compare Fig. S4 for additional experimental sets. Color scheme as in Fig. 7

As highlighted above, a particular advantage of 5D over 4D amide-to-amide experiments is the use of a shift triplet as a source for assignment, facilitating matchmaking with side-chain resonances. This concerns Cβ resonances, obtained via hcaCBcaNH type experiments, or the set of sidechain carbons, obtained via S2B experiments. In addition to sidechain carbons, S2B experiments can be used for the assignment of protons in sidechains of protonated samples. This is highly sought for structure calculation, chemical-shift perturbations outside the set of backbone shifts, and other analyses of the protein (Andreas et al. 2016; Stanek et al. 2016; Vasa et al. 2019). Recently we demonstrated that sidechain protons can be reliably assigned using a 5D S2B, in particular the combined 5D HCCNH/4D HCCH experiment (Klein et al. 2022b). Whereas a similar approach could be used for partially protonated sidechains (for example from RAP (Asami and Reif 2013) or iFD labeling (Medeiros-Silva et al. 2016), we demonstrated this for non-deuterated samples using high spinning frequencies (> 100 kHz) for narrow proton linewidth. The 5D HCCNH experiment presents essentially the same HN bulk sensitivity as the 3D hcaCBcaNH, but one HN signal detected in the bulk carries cumulative magnetization stemming from a multitude of individual H/C moieties. In congruence with the above thought of streamlining backbone assignments in small proteins by a minimal set of higher-dimensionality experiments, here we tested whether a full (backbone and sidechain) assignment can also be leveraged by a minimal number of (accordingly higher-complexity) experiments. For expansion of resonance assignment to the sidechain H/C moieties we replaced the hcaCBcaNH experiment in the sets described above with the 5D HCCNH. This demands more measurement time (The S2B experiment was recorded in a 0.7 mm rotor for 6 days.), but the set allows coverage of all but the carbonyl resonances from only two experiments (Fig. 8A) within cumulative measurement time of 8 days at 700 MHz (or 8.5 d when an hCONH is included for added CO assignments). These minimal sets of experiments combine data from different preparations, namely the deuterated/amide-back-exchanged sample spun at 55 kHz for the backbone experiment(s) and the fully protonated sample spun at 110 kHz for the S2B. The assignments are successful even though we did not take specific care of adjusting the temperature to a common value (25 °C in the 1.3 mm rotor vs. 20 °C in the 0.7 mm rotor), which entails slight chemical-shift deviations between the experiments. For comparison, the set of established 3D experiments of an equivalently complete strategy would at least incorporate hCANH/hCAcoNH, hCONH/hCOcaNH, hcaCBcaNH, HNHA, hCCH, and HCcH, i.e., eight experiments and a larger extent of manual handling, including referencing the individual experiments and substantial efforts of picking the many peaks in crowded 3D side-chain spectra. Following a manual assignment strategy with exclusively the two 5D experiments (recording the 5D HCCNH with a simultaneous 4D HCCH, as shown in Klein et al. 2022b), 88% of all resonances, excluding the side-chain protons of aromatic residues, can be assigned from two 5Ds. (Mixing between aliphatic and aromatic carbons still represents a hurdle and has only recently been overcome in solid-state NMR for uniformly labelled proteins (Ahlawat et al. 2023).) In this case, an additional 3D hCANH experiment served as a base for reconstruction but could in principle be replaced by the H/N/CA information (the strongest peaks) already intrinsically included in 5D S2B experiment.

While the manual sidechain assignment strategy remains straightforward when using the 5D HCCNH experiment (The complete set of sidechain H/C peaks is obtained in a 2D hCH type spectrum per residue, assuming that no overlap in the hCANH 3D space is present.), the application of FLYA can accelerate the process further, with no or little compromise for the assignment quality, based on the results for the backbone assignments. From FLYA assignments, based on the same minimal set as for the manual assignments (the 5D HNcoCANH, 5D HCCNH, and the hCANH as a base for SMFT), 95% of the strongly assigned resonances are identical with the manual assignments (dark green) and 4% are erroneous (Fig. 8B; Table 3). An output with this level of quality is sufficiently good for structure calculation without further manual corrections (Klein et al. 2022b). When using the *4D* HNNH version for backbone sequential connectivities, only slightly lower assignment success is achieved (Fig. 8C; Table 3). This is due to the fact that the S2B experiment is the bottleneck and a slightly lower backbone assignment fidelity does not lead to a further decrease of the success rate. If sufficient resolution in the H/N correlation is warranted, the S2B and amide-to-amide experiments combined here can also both be acquired as 4Ds, i.e., 4D HNcocaNH and 4D HCcNH (Fig. 8D; Table 3). Despite the low molecular weight and high β-sheet content of the SH3-domain, the hNH spectrum does comprise regions with signal overlap (Fig. 1) and hence ambiguities in the backbone assignment are obtained. However, given the intrinsic gain in sensitivity by $\surd 2$, the detection of the weakest resonances within the large dynamic range is facilitated. As such, even under the conditions chosen here (4.25 days and 6 days in the case of the 4D HCcNH and 5D HCCNH experiment, respectively), around 13% more resonances are found using the 4D HCcNH than in the 5D HCCNH (when used on its own, ignoring the interleaved 4D HCCH), upon manual assignment. The additional assignments generally refer to weak resonances, emphasizing sensitivity as the critical aspect here. However, when processed in conjunction with the simultaneous 4D HCCH experiment from orphaned magnetization, in the framework of *manual* assignment, the 5D HCCNH approach facilitates even slightly more assignments than the 4D HCcNH version. Conversely, as reported previously (Klein et al. 2022b), the poor chemical shift redundancy in the 4D HCCH and the associated many assignment possibilities lead to no further improvements in the *automated* assignment process with FLYA, such that the FLYA results shown in Fig. 8 and S4 are always obtained without the 4D HCCH experiment.

**Table 3:** Completeness and correctness of the individual FLYA runs depicted in Fig. 8 above. Analysis as in Table 1. While runs 8B to 8D perform similarly in terms of overall assignment, the combination of 5D backbone and 5D S2B experiments (8B) yields the highest number of correct and “strong” assignments. The missing signals in the benchmark 5D HNcoCANH (36 h) cause a decrease in the overall assignments made by FLYA as can be seen from the higher number of black rectangles in Fig. 8E.

Run	Correct and strong	Overall strong	All assigned
8B	301	317	422
8C	258	289	433
8D	278	333	432
8E	263	292	392

When the 5D HCCNH S2B (without the 4D HCCH peak list) is combined with a 4D backbone experiment, only 89% of all strong assignments are correct (Fig. 8C; Table 3), which rate is further decreased to 83% when both experiments are taken into account as 4Ds (Fig. 8D). Even when supplementing the FLYA runs with an additional hCANH peak list, the trends are maintained (see Fig. S4, 88% for 4D/4D and 91% for 4D/5D). Additionally, we assessed the assignment success of the minimal set involving two 5D spectra when the HNcoCANH is recorded for only 36 h (8 scans, Fig. 8E). The assignment success (90% correct assignments, 8% erroneous) lies in between what is obtained when acquiring this backbone experiment longer (Fig. 8B) and slightly above the FLYA assignment involving the 4D HNNH experiment instead (Fig. 8C). Despite the increase in mismatching assignments, a remarkable extent of backbone assignments is still obtained through automated assignment from these two data sets. Even though in this case, the 5D versions do not outcompete the computational use of 4D sets by much, the results still show that not only manual assignments can benefit from 5D data, but even in automated routines for combined backbone and sidechain assignments, the fifth dimension can be of advantage. The FLYA results in total highlight that high degrees of assignments (backbone and sidechains) of proteins can be obtained with minimal manual expert user interference from only *two* (highly complementary, higher-dimensionality) experiments.

## Discussion

The above data suggests that even for small to mid-range molecular-weight targets, the traditional assignment process, based on pairs of complementary carbon match-making experiments, can realistically be alleviated by replacing the numerous spectra of lower information content by fewer, higher-information-content experiments. The compressed information content addresses multiple hurdles associated with the assignment process, in particular upon manual expert user assignments. The number of spectra that the user has to oversee simultaneously, display and maintain their orientation within, is much lower. Finding the matching resonance of the next/previous neighbor by combined processing of multiple strips by traditional means is usually not straightforward. In particular, when a complementary pair of 3Ds derives from partially overlapping H/N peaks, consolidating 6 spectra to validate each one of the numerous trials necessary with the usual degrees of carbon shift similarity easily becomes confusing. Instead, from fewer, higher-dimensionality experiments, the user benefits from a direct identification of the next/previous amide coordinates from a simple 2D plane out of a single, preassembled stack directly linked to residue type information. Similarly, referencing of spectra to each other is facilitated. Even though sensitivities of the higher-dimensionality experiments are still lower than the average 3D experiment, the measurement times for a *set* of experiments are highly competitive, as very insensitive experiments like the hcaCBcacoNH are unnecessary. Current developments, e.g., in the field of optimal-control NMR, hold promise that the sensitivity of higher-dimensionality experiments can be further (drastically) increased in the future (Blahut et al. 2022) and are currently investigated by us. Within the boundaries of the above assessments, automated assignments seem to be performing at least similarly well, comparing carbon-match-making and higher-dimensionality direct-concatenation approaches, but (for this sample) also demonstrating the performance of 4D experiments as maintained alternatives to the 5Ds. We realize that a first implementation of the processing routines is associated at current with an activation barrier, as the way of data handling feels different to long-established procedures, which combats the simplification of the assignment process itself. We hope that a more broadband implementation and standardization of 4D and 5D processing into increasingly user-friendly software packages will overcome this hurdle for future users and allow an easy access to the benefits of higher-dimensionality data for the next generation of solid-state NMR spectroscopists.

## Conclusion

Here we have demonstrated the very general value of 5D amide-to-amide experiments for facilitated assignment of micro-crystalline or quasi-crystalline proteins including low to mid-range molecular weight. Due to their low level of ambiguity upon sequential correlations and high-fidelity match-making between sequential information and information on residue type, the higher-dimensionality approaches effectively reduce ambiguity upon peak assignment with less or at least comparable measurement times relative to traditional triple-resonance experiments. In addition, the low number of complementary experiments with compressed information content each minimize the combinatorial and organizational effort on the user side and further facilitate the assignment process by eradication of human errors in practice. As such, the experiments used here provide competitive performance for backbone and side-chain assignments over traditional techniques and can be effectively combined with automated assignment algorithms. Once the pipelines for setup and processing of higher-dimensionality spectra have been established as routine tools in a given research environment, these approaches can streamline manual and automated assignment of future solid-state NMR targets and hence leverage one of the currently most time-consuming and cumbersome steps of bio-ssNMR studies for new target systems.

### Electronic supplementary material

Below is the link to the electronic supplementary material.

Supplementary Material 1
