# Supplementary material for: 5D solid-state NMR spectroscopy for facilitated resonance assignment
Source: J Biomol NMR. 2023 Nov 9;77(5-6):229–45. doi: 10.1007/s10858-023-00424-5 (PMC10687145; doi:10.1007/s10858-023-00424-5)
Supplement: Supplementary file 1 — Supplementary Material 1 [file 10858_2023_424_MOESM1_ESM.pdf]

Support Information for 5D solid-state NMR spectroscopy for facilitated resonance assignment

Alexander Klein, Suresh K. Vasa, Rasmus Linser

Acquisition parameters

Table S1: CP conditions for the 5D experiments and the 3D experiments used as base

**3D hCANH**				
**Hard pulses**	µs	RF / kHz		
^{1}H	1.5	166.7		
^{15}N	4.9	51.02		
^{13}C	3.6	69.44		
**H-C CP**		RF ^{1}H / kHz	RF ^{15}N / kHz	RF ^{13}C / kHz
Contact time	2000	30.93		31.74
Shape		100-50 tang.		rectangular
**Ca-N CP**		RF ^{1}H / kHz	RF ^{15}N / kHz	RF ^{13}C / kHz
Contact time	11000		36.88	17.54
Shape			90-100 ramp	rectangular
**N-H CP**		RF ^{1}H / kHz	RF ^{15}N / kHz	RF ^{13}C / kHz
Contact time	400	19.51	41.38	
hape		50-100 tang.	90-100 ramp	

**5D HNcoCANH**				
**Hard pulses**	µs	RF / kHz		
^{1}H	1.5	166.7		
^{15}N	4.9	51.02		
^{13}C	3.6	69.44		
**N-H CP**		RF ^{1}H / kHz	RF ^{15}N / kHz	RF ^{13}C / kHz
Contact time	150	19.51	41.38	
Shape		100-50 tang.	90-100 ramp	
**N-CO CP**		RF ^{1}H / kHz	RF ^{15}N / kHz	RF ^{13}C / kHz
Contact time	11000		39.06	16.56
Shape			90-100 ramp	rectangular
**CO-Ca BSHCP**		RF ^{1}H / kHz	RF ^{15}N / kHz	RF ^{13}C / kHz
Contact time	6900			23.67
Shape				80-100 ramp

**Ca-N CP**		RF ^{1}H / kHz	RF ^{15}N / kHz	RF ^{13}C / kHz
Contact time	11000		38.17	17.54
Shape			90-100 ramp	rectangular
**N-H CP**		RF ^{1}H / kHz	RF ^{15}N / kHz	RF ^{13}C / kHz
Contact time	150	19.51	41.38	
Shape		50-100 tang.	90-100 ramp	

**3D hCONH**				
**Hard pulses**	µs	RF / kHz		
^{1}H	1.75 µs	143		
^{15}N	3.4 µs	73.5		
^{13}C	2.4 µs	104		
**H-Ca CP**		RF ^{1}H / kHZ	RF ^{13}C / kHz	RF ^{15}N / kHz
Contact time	2900 µs	18.7	40.7	
Shape		100-50 tang.	rectangular	
**Ca-N CP**		RF ^{1}H / kHZ	RF ^{13}C / kHz	RF ^{15}N / kHz
Contact time	7500 µs		19.9	34.4
Shape			rectangular	90-100 ramp
**N-H CP**		RF ^{1}H / kHZ	RF ^{13}C / kHz	RF ^{15}N / kHz
Contact time	1000 µs	17.9		36.8
Shape		50-100 tang.		rectangular

**5D HNcaCONH**				
**Hard pulses**	µs	RF / kHz		
^{1}H	1.75 µs	143		
^{15}N	3.4 µs	73.5		
^{13}C	2.4 µs	104		
**H-Ca CP**		RF ^{1}H / kHZ	RF ^{13}C / kHz	RF ^{15}N / kHz
Contact time	2900 µs	18.7	40.7	-
Shape		100-50 tang.	rectangular	
**Ca-N CP**		RF ^{1}H / kHZ	RF ^{13}C / kHz	RF ^{15}N / kHz
Contact time	7500 µs	-	19.9	34.4
Shape			rectangular	90-100 ramp
**N-H CP**		RF ^{1}H / kHZ	RF ^{13}C / kHz	RF ^{15}N / kHz
Contact time	1000 µs	17.9	-	36.8
Shape		50-100 tang.		rectangular

Table S2: Acquisition parameters of the 5D experiments and their 3D base experiments recorded

**5D HNcoCANH**					
	F5	F4 (^{15}N)	F3 (^{15}N)	F2 (^{1}H)	F1(^{13}Ca)
FID size	2048	54	54	46	50
SW / ppm	29.75	39.15	39.15	6.61	35.49
Increment size / us		360	360	216	160
Aq. Time / ms	49.15	9.72	9.72	4.97	4.00
Scans	24	Exp. time: 6.5 days			
NUS points	2048	(0.5%)			
Truncated data					
Scans	8				
NUS pts. (exp.time)	2048 (52 h), 1536 (39 h), 1024 (26 h), 512 (13 h)				
	256 (6.5 h), 192 (5 h), 64 (1.5 h)				
Short recording					
Scans	8	Exp. time: 36 h			
NUS points	1805	(0.4%)			
**5D HNcaCONH**					
	F5	F4 (^{15}N)	F3 (^{15}N)	F2 (^{1}H)	F1(^{13}CO)
FID size	1024	96	96	64	80
SW / ppm	15.51	30.6274	30.6274	4.9575	15.7716
Increment size / us		460	460	288	360
Aq. Time / ms	47.1	22.08	22.08	11.52	10.08
Scans	16	Exp. time: ca. 4d			
NUS points	1920	(0.1%)			
**3D hCONH**					
	F3	F2 (^{15}N)	F1		
FID size	2048	192	152		
SW / ppm	29.74	39.1350	21.0262		
Increment size / us		360	270		
Aq. Time / ms	49.1	34.56	20.52		
Scans	16	Exp. time: ca. 9h			
NUS points	1024	(14.0%)			
**3D hCANH**					
	F3	F2 (^{15}N)	F1		
FID size	2048	160	112		
SW / ppm	29.75	39.15	39.44		
Increment size / us		360	144		
Aq. Time / ms	49.1	29.88	7.92		
Scans	16	Exp. time: 12.5 h			
NUS points	1024	(22.9%)			

Assessment of diagonal peaks in INEPT based pulse sequences

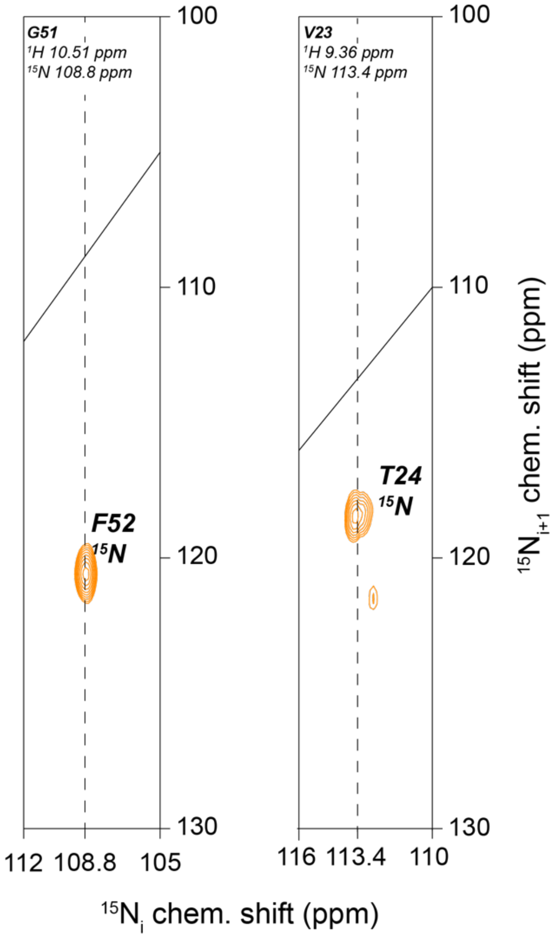

Fig. S1: *Strip plots of the 3D hNcocaNH experiment recorded on a fully protonated sample of the SH3-domain of chicken α-spectrin. The same correlations as in Fig. 4 (main text) are shown. The strips are displayed for the HN coordinates of a residue i (G51 and V23) correlated with the ^{15}N chemical shift of residue i+1 (F52 and T24). For none of the cross peaks found in the spectrum a diagonal peak is found. The spectrum was recorded at a ^{1}H Larmor frequency of 700 MHz for 20.5 h using 32 scans with a t_{max} of 14.7 ms in the i ^{15}N dimensions, and 7.2 ms in the i+1 ^{15}N dimension.*

Signal-to-noise comparison with a 3D hCANH of the same sample

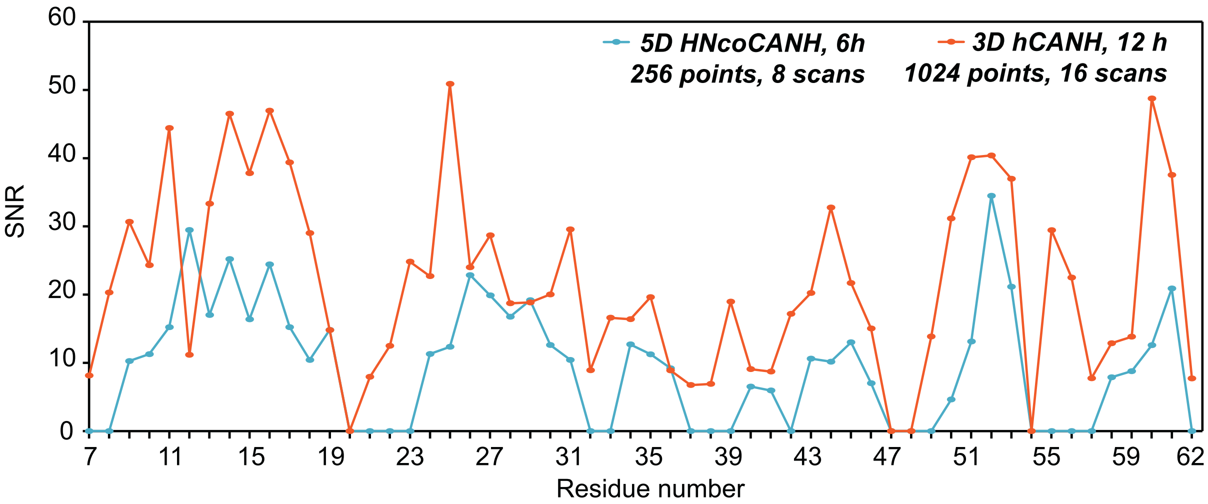

Fig. S2: *SNR per residue for a 3D hCANH of the same sample (also recorded as NUS experiment) using 1024 points and 16 scans (red). In addition, the graph depicts again the signal-to-noise ratio of the 5D HNcoCANH truncated to 256 NUS points and recorded with 8 scans (blue). This truncated data set (Main text Fig. 6) is the scenario that comes closest to the hCANH with respect to its effective experimental time and with 75% of the peaks may represent a minimal acceptable performance. The average SNR of 24 reached for the hCANH under the above conditions might serve as guideline to estimate the factor in measurement times for the 5D for future samples that a 3D hCANH (or similar) has been successfully recorded for. If it takes n-times longer to reach a SNR of around 30 for another sample, the number of NUS points in the 5D (with ns = 8) will need to be chosen at least n-times higher than what is shown for the different levels of performance for SH3 in Main Text Fig. 6. SNR and noise levels were calculated in CCPNmr v3.2 and should generally be seen as estimates as NUS is used in all spectra presented. The SMFT procedure for the 5D processed as 2D planes might additionally affect the effective signal-to-noise but noise levels in all spectra were generally found to be in good agreement with the noise levels calculated by SSA after reconstruction.*

Automated resonance assignment using FLYA

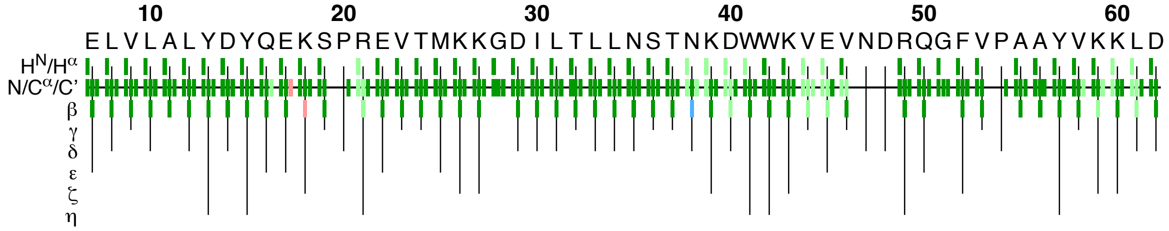

Fig. S3: *FLYA assignments obtained from a 5D HNcoCANH recorded for 36 h, a 3D hcaCBcaNH, a hNH, and a 3D hCONH experiment. The peak lists are equivalent to the ones used for the results shown in Fig. 6E augmented by the 3D hCONH to fill in the missing CO resonances. 100 independent FLYA runs were combined into a consensus assignment. The tolerances are 0.05 ppm for ^{1}H, and 0.5 ppm for ^{13}C and ^{15}N, respectively. Despite a slight reduction in strong identical assignments, no more erroneous assignments are made.*

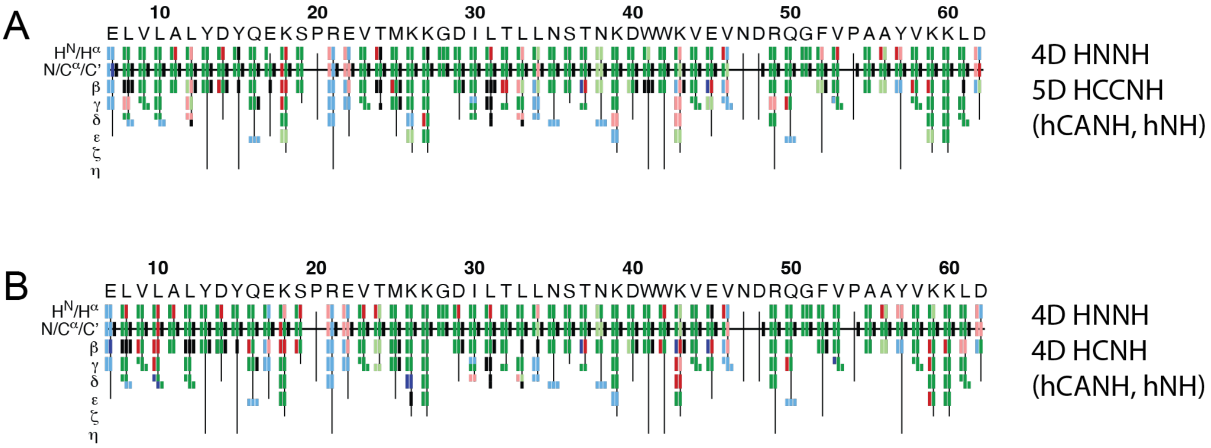

Fig. S4: *Side-chain and backbone assignments obtained with FLYA as in the main text Fig. 7. The runs using the 4D HNcocaNH experiment were supplemented with a 3D hCANH experiment. The tolerances are 0.1 ppm for ^{1}H, 0.8 ppm for ^{13}C, and 0.5 ppm for ^{15}N, respectively. Despite a slight reduction in strong identical assignments, no more erroneous assignments are made.*

Table S3: *Completeness and correctness of the individual FLYA runs depicted in Fig. S4 above. Analysis as in Table 2 and 3 (main text). The number of overall assigned resonances remains relatively constant, while more overall strong and correct assignments are made when the 3D hCANH is incorporated. The certainly traces back to the improved redundancy of the side-chain Ca resonances with the backbone through the hCANH experiment.*

Run	Correct and strong	Overall strong	All assigned
S3A	281	308	430
S3B	304	347	441
